# National Cancer Grid initiative for electronic medical records, India

**DOI:** 10.2471/BLT.24.292230

**Published:** 2025-04-01

**Authors:** C S Pramesh, Rizwan Koita, Manju Sengar, Nikesh Shah, Anthony Vipin Das, Prakash Nayak, Kiran Anandampillai, Prathamesh Pai, Amrut Kadam, Indranil Mallick, Prabhat Bhargava, Prasanth Penumadu, Chandran K Nair, Bibhuti Borthakur, M Aarish, Geetu Bagri, Sarbani Ghosh-Laskar, Anil Tibdewal, Latha Balasubramani, Abhishek Jain, Aditya Jandial, Gagan Prakash, Nilesh Teli, Smita Kayal, Surabhi Goel, Krupa Mayekar, Priya Ranganathan, Vandana Agarwal, Madhavi Shetmahajan, Reshma Ambulkar, Jayita Deodhar, Aparna Chatterjee, Mukkesh Bansal

**Affiliations:** a Tata Memorial Hospital, Tata Memorial Centre, Homi Bhabha National Institute, National Cancer Grid, E. Borges Road, Parel, Mumbai 400012, India.; b Koita Foundation, Koita Centre for Digital Oncology, Mumbai, India.; c National Cancer Grid, Koita Centre for Digital Oncology, Mumbai, India.; d Fernandez Hospital Education and Research Foundation, Hyderabad, India.; e iDrishti Eye Hospitals, Bangalore, India.; f Punyashlok Ahilyadevi Holkar Head and Neck Cancer Institute of India, Mumbai, India.; g Victoria Hospital, Bangalore Medical College, Bangalore, India.; h Tata Medical Center, Kolkata, India.; i Sri Venkateswara Institute of Cancer Care and Advanced Research, Tirupati, India.; j Malabar Cancer Centre, Thalassery, India.; k B Borooah Cancer Institute, Guwahati, India.; l G Kuppuswamy Naidu Memorial Hospital, Coimbatore, India.; m Swasth Alliance, Bangalore, India.; n Homi Bhabha Cancer Hospital and Research Centre, Mullanpur, India.; o Jawaharlal Institute of Postgraduate Medical Education and Research, Puducherry, India.

## Abstract

**Problem:**

Inefficient workflows, incomplete data and lack of interoperability can hinder the uptake of electronic records systems, challenges particularly relevant in cancer treatment with its complex longitudinal and multidisciplinary nature. Further, products developed in high-income countries are not designed for compatibility with the workflows of low- and middle-income countries, which face additional issues of cost.

**Approach:**

We evaluated centres with different resources and geographical locations to develop the requirements of our product. We published an invitation to potential vendors, evaluated submitted product development bids and enlisted six vendors. Our subcommittees developed workflow modules and templates, ensured interoperability and developed key performance indicators.

**Setting:**

The National Cancer Grid, a network of more than 360 cancer centres in India, assembled a team of experienced oncologists and digital health experts to develop electronic medical records products with specialized oncology capabilities.

**Relevant changes:**

Our collaboration between clinical and technical experts led to the development of six new, high-quality and interoperable products, compliant with the varying needs and resources of hospitals. We supported more than 20 centres with procurement and adoption through partial funding and technical assistance.

**Lessons learnt:**

In developing product requirements, we gained an understanding of the challenges faced by hospitals in implementing such systems; by inviting vendors to submit a product development bid, we ensured that the product development cost was borne by the vendor and not hospitals; and by monitoring user feedback, we can continue to address issues raised by health workers and encourage the adoption of electronic medical records.

## Résumé

**Problème:**

Des procédures de travail inefficaces, des données incomplètes et un manque d’interopérabilité sont susceptibles d’entraver la transition vers les dossiers médicaux électroniques. Ces défis sont particulièrement pertinents dans le traitement du cancer, vu sa nature complexe, longitudinale et multidisciplinaire. En outre, les solutions mises au point dans les pays à revenu élevé ne sont pas nécessairement compatibles avec les procédures de travail des pays à revenu faible ou moyen, qui sont confrontés à des problèmes supplémentaires liés au coût.

**Approche:**

Nous avons évalué des centres disposant de ressources et de situations géographiques différentes pour définir les exigences de notre solution. Nous avons publié un appel d’offres à l’intention de fournisseurs potentiels, évalué les offres de création de solutions soumises et retenu six fournisseurs. Nos sous-comités ont élaboré des modules et des modèles de procédures de travail, veillé à l’interopérabilité et créé des indicateurs de performance clés.

**Environnement local:**

Le National Cancer Grid, réseau qui rassemble plus de 360 centres de cancérologie en Inde, a réuni une équipe d’oncologues expérimentés et d’experts en santé numérique pour élaborer des solutions de dossiers médicaux électroniques avec des capacités spécialisées en oncologie.

**Changements significatifs:**

Notre collaboration entre les experts cliniques et techniques a permis la mise au point de six nouvelles solutions de haute qualité et interopérables, conformes aux différents besoins et ressources des hôpitaux. Nous avons aidé plus de 20 centres à acquérir et à adopter ces solutions grâce à un financement partiel et à une assistance technique.

**Leçons tirées:**

En définissant les exigences relatives aux solutions, nous sommes parvenus à comprendre les difficultés rencontrées par les hôpitaux dans la mise en œuvre de ces systèmes; en invitant les fournisseurs à soumettre une offre de développement de solution, nous avons veillé à ce que ce soit le fournisseur et non les hôpitaux qui supporte le coût de développement de la solution; et en analysant les réactions des utilisateurs, nous sommes en mesure de continuer à traiter les questions soulevées par les travailleurs de la santé et à encourager l’adoption des dossiers médicaux électroniques.

## Resumen

**Situación:**

Los flujos de trabajo ineficientes, los datos incompletos y la falta de interoperabilidad pueden obstaculizar la adopción de sistemas de historia clínica electrónica, desafíos especialmente relevantes en el tratamiento del cáncer debido a su naturaleza compleja, longitudinal y multidisciplinar. Asimismo, los productos desarrollados en países de ingresos altos no están diseñados para ser compatibles con los flujos de trabajo de los países de ingresos bajos y medianos, que enfrentan, además, problemas adicionales relacionados con el coste.

**Enfoque:**

Se evaluaron centros con distintos niveles de recursos y ubicaciones geográficas para definir los requisitos del producto. Se publicó una convocatoria dirigida a posibles proveedores, se evaluaron las propuestas de desarrollo presentadas y se seleccionaron seis proveedores. Los subcomités elaboraron módulos de flujo de trabajo y plantillas, garantizaron la interoperabilidad y desarrollaron indicadores clave de rendimiento.

**Marco regional:**

La *National Cancer Grid*, una red de más de 360 centros oncológicos en la India, conformó un equipo de oncólogos experimentados y expertos en salud digital para desarrollar productos de historia clínica electrónica con funcionalidades especializadas en oncología.

**Cambios importantes:**

La colaboración entre profesionales clínicos y expertos técnicos permitió el desarrollo de seis productos nuevos, interoperables y de alta calidad, adaptados a las necesidades y recursos variables de los hospitales. Más de 20 centros recibieron apoyo para la adquisición e implementación de estos productos mediante financiación parcial y asistencia técnica.

**Lecciones aprendidas:**

Durante la definición de los requisitos del producto, se obtuvo una comprensión más profunda de los desafíos que enfrentan los hospitales al implementar estos sistemas. Al invitar a los proveedores a presentar propuestas de desarrollo, se consiguió que los costes fueran asumidos por estos y no por los hospitales. Asimismo, el seguimiento de la retroalimentación de los usuarios permite seguir abordando los problemas señalados por el personal sanitario y fomentar la adopción de las historias clínicas electrónicas.

## ملخص

المشكلة إن عمليات سير العمل غير الفعّالة، والبيانات غير المكتملة، ونقص التوافق التشغيلي، يمكنها أن تعيق تنفيذ أنظمة السجلات الإلكترونية، وهي تحديات ذات صلة كبيرة بعلاج السرطان مع طبيعته المعقدة وتعدد تخصصاته. علاوة على ذلك، فإن المنتجات المُطوّرة في الدول ذات الدخل المرتفع غير مُصمّمة للتوافق مع عمليات سير العمل في الدول ذات الدخل المنخفض والدخل المتوسط، والتي تواجه تحديات إضافية تتعلق بالتكلفة.

الأسلوب قمنا بتقييم مراكز ذات موارد ومواقع جغرافية مختلفة لتطوير متطلبات منتجنا. قمنا بنشر دعوة للموردين المحتملين، وتقييم عروض تطوير المنتج المُقدّمة، وضم ستة موردين. قامت اللجان الفرعية لدينا بتطوير وحدات وقوالب سير العمل، وضمنت التوافق التشغيلي، ووضعت مؤشرات أداء رئيسية.

المواقع المحلية قامت الشبكة الوطنية للسرطان، وهي شبكة تضم أكثر من 360 مركزًا للسرطان في الهند، بتجميع فريق من أطباء الأورام ذوي الخبرة وخبراء الصحة الرقمية، لتطوير منتجات للسجلات الطبية الإلكترونية ذات قدرات متخصصة في علم الأورام.

التغيّرات ذات الصلة أدى التعاون لدينا بين الخبراء الإكلينيكيين والتقنيين إلى تطوير ستة منتجات جديدة عالية الجودة وقابلة للتشغيل البيني، ومتوافقة مع الاحتياجات والموارد المتنوعة للمستشفيات. وقد دعمنا أكثر من 20 مركزًا في عمليات الشراء والاعتماد من خلال التمويل الجزئي والمساعدة التقنية.

الدروس المستفادة عند تطوير متطلبات المنتج، اكتسبنا فهمًا للتحديات التي تواجهها المستشفيات في تنفيذ هذه الأنظمة؛ ومن خلال دعوة الموردين لتقديم عروض تطوير المنتج، تحققنا من أن يتحمل المورد وليس المستشفيات تكلفة تطوير المنتج؛ ومن خلال رصد ملاحظات المستخدمين، يمكننا مواصلة معالجة القضايا التي يثيرها العاملون في مجال الصحة، وتشجيع اعتماد السجلات الطبية الإلكترونية.

## 摘要

**问题:**

工作流程效率低下、数据不完整和互操作性缺乏可能会阻碍电子病历系统的使用，这些挑战在癌症治疗领域尤为突出，这是因为癌症治疗具有复杂的纵向性和多学科性。此外，高收入国家开发的产品并未针对低收入和中等收入国家的工作流程进行兼容性设计，因此此类中低收入国家还面临着更多成本问题。

**方法:**

我们对拥有不同资源且位于不同地理位置的中心进行评估以确定我们的产品要求。我们向潜在供应商发布了投标邀请书，对供应商提交的产品开发投标书进行了评估并选出了六家供应商。我们的小组委员会制定了工作流程模块和模板，确保了互操作性并设定了关键业绩指标。

**当地状况:**

作为一个涵盖了 360 多个肿瘤治疗中心的网络，印度国家癌症网格组建了一支由经验丰富的肿瘤医生和数字卫生专家组成的团队，以开发专门为肿瘤科设计的电子病历产品。

**相关变化:**

在临床专家与技术专家的通力合作下，我们开发出了六款高质量且具有互操作性的新产品，从而可满足具有不同资源条件的各医院的不同需求。我们通过部分供资和提供技术援助帮助 20 多个中心完成了产品的采购和采用。

**经验教训:**

在确定产品要求的过程中，我们了解了各医院在实施此类系统过程中所面临的挑战；通过邀请供应商提交产品开发投标书，我们确保了产品开发成本由供应商而非医院承担；且通过监测用户反馈，我们可以继续解决卫生工作者提出的问题并鼓励各医院采用电子病历。

## Резюме

**Проблема:**

Неэффективные рабочие процессы, неполные данные и недостаточная совместимость могут негативно влиять на распространение систем электронной документации, и эти проблемы особенно остро ощущаются в сфере онкологии, где лечение часто бывает длительным, сложным и мультидисциплинарным. Кроме того, продукция, разработанная в странах с высоким уровнем дохода, не предусматривает совместимости с рабочими процессами, принятыми в странах со средним и низким доходом, что приводит к дополнительным затратам.

**Подход:**

Авторы оценили работу центров разной степени оснащенности в различных географических регионах, чтобы разработать требования к нашей продукции. Был объявлен конкурс среди потенциальных поставщиков, была проведена оценка заявок на разработку продукта, и были отобраны шесть поставщиков. Наши подкомитеты разработали модули и шаблоны рабочих процессов с обязательным требованием взаимной совместимости и определили ключевые показатели эффективности работы.

**Местные условия:**

Национальная сеть по борьбе с раком, которая включает более 360 онкологических центров в Индии, собрала команду опытных онкологов и специалистов по цифровой медицине, чтобы разработать средства электронного документооборота с учетом специальных требований, предъявляемых онкологическими учреждениями.

**Осуществленные перемены:**

Благодаря сотрудничеству клинических и технических специалистов были разработаны шесть новых высококачественных и полностью взаимно совместимых технических средств с учетом различий в их финансовых и технических возможностях. Для приобретения и внедрения данных разработок более 20 центрам была оказана частичная финансовая помощь и техническая поддержка.

**Выводы:**

Разработка продукции позволила лучше понять проблемы, с которыми сталкиваются лечебные учреждения при внедрении подобных систем. Приглашение поставщиков на конкурсной основе позволило возложить расходы по разработке на поставщиков, а не на лечебные учреждения, и отслеживание реакции пользователей дало возможность устранить отмеченные ими недостатки и поощрило внедрение систем электронной медицинской документации.

## Introduction

The implementation of well-designed electronic medical records can enhance the delivery of high-quality care and improve patient outcomes through better documentation, adherence to evidence-based treatment guidelines, reduction in medication errors and improved communication between health workers.^1^ Cancer treatment, with its complex longitudinal and multidisciplinary nature across the continuum from diagnosis to multimodality treatment and survivorship, requires reliable documentation that is accessible to all stakeholders; electronic medical records are therefore even more relevant in oncology.^2^ Data from electronic records can also enable real-world studies and facilitate quality improvement in cancer treatment.^3^

The benefits of electronic medical records in oncology can however remain elusive because of inefficient workflows, design and interface issues, incomplete data availability, difficulties with data retrieval and, importantly, a lack of interoperability,^4^ even in high-income countries. While some initiatives have worked towards addressing these challenges, the implementation of electronic records remains limited.^5^

The role of electronic records in contributing to the delivery of high-quality care is even more important in low- and middle-income countries, which have fewer resources and a smaller workforce compared with high-income countries.^6^ However, existing electronic medical records solutions developed in high-income countries are (i) expensive;(ii) require considerable investment in infrastructure, training and time from already overworked clinicians; and (iii) do not reflect the process flows of low- and middle-income countries, limiting their adoption.^7^^,^^8^ Therefore, affordable solutions that consider workflows, resource availability, interoperability and simple data capture are needed for low- and middle-income countries to benefit from electronic records. 

We describe our initiative to develop and deploy electronic medical records products applicable in both small and large hospitals, and that benefit all stakeholders without duplication of efforts in data collection or extraction. Our initiative aims to provide data ownership to patients; enhance the efficiency of clinicians and reduce the risk of errors in care; enable the monitoring of quality of care; and facilitate seamless, decentralized and evidence-based cancer care. 

## Setting

The National Cancer Grid, a network of more than 360 cancer centres and institutes across India, was established to ensure equitable cancer care in India.^9^ Approximately 850 000 new cancer cases (60% of the 1.4 million new cancer cases in India annually, according to unpublished National Cancer Grid data) are treated annually across the member centres. In a 2022 survey of member organizations to identify the requirements of oncologists in the adoption of digital technology to enhance cancer care, 80% of those who responded (81/101) prioritized electronic medical records. Under the guidance of the Koita Centre of Digital Oncology,^10^ the National Cancer Grid has therefore been focusing on the development and adoption of user-friendly, interoperable electronic records products that meet the digital health standards developed by the Ayushman Bharat Digital Mission.^11^ The key elements of such products include the automation of workflows, standardized data capture, strong data security and protection features, compliance with the digital data protection act and consent-based health information exchange.

## Approach

**Fig. 1:**
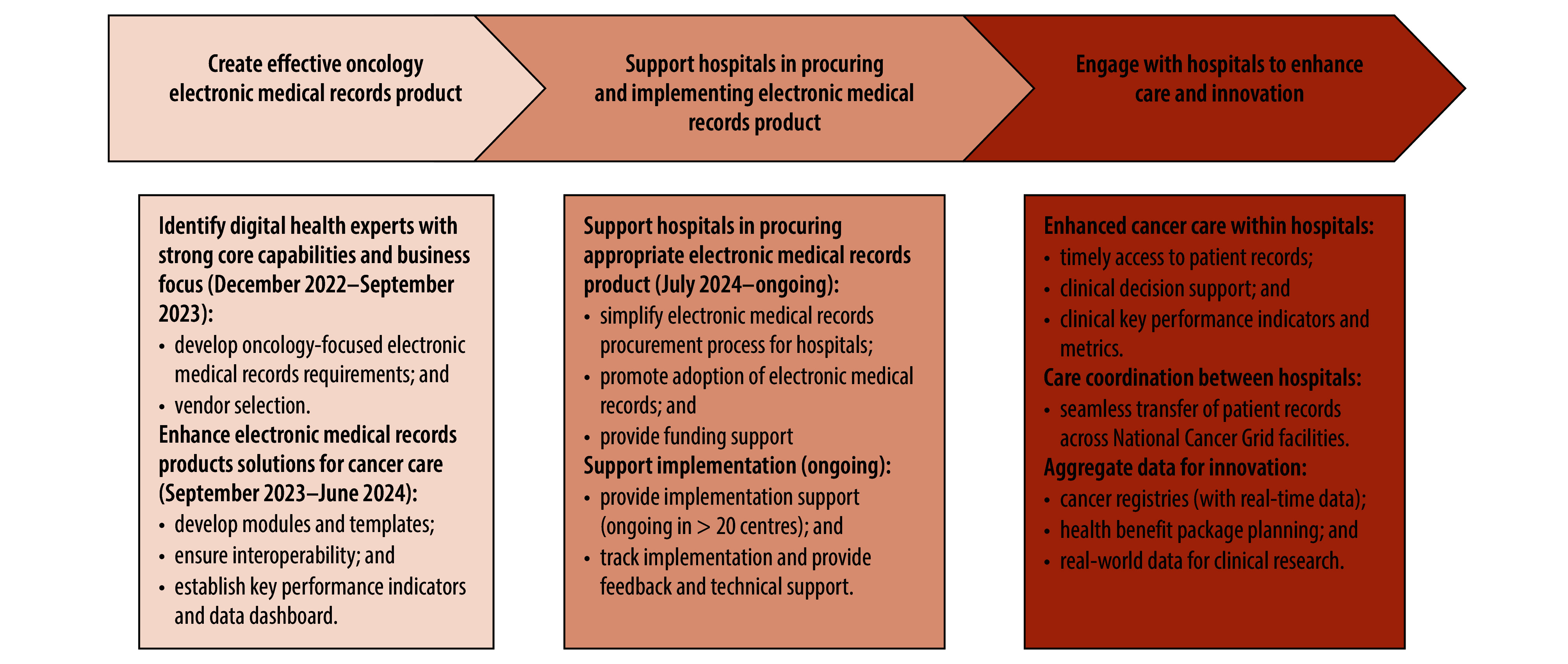
Stages of the development and deployment of electronic medical records by the National Cancer Grid, India

Fig 1. shows the development and deployment phases of the project. 

### Development of requirements

To define the requirements of electronic medical records products in oncology, we collated information on potential areas for improvement from customer reviews and user feedback. Our core team of experienced oncologists from National Cancer Grid centres with an understanding of digital technology, as well as technical experts with experience in digital health solutions, developed these requirements. Our team evaluated representative centres from different geographical regions and with different levels of resources to understand the varying process workflows, availability of technology support and existing hospital management information system (if any). We then performed a gap assessment to identify the organizational requirements for electronic medical records deployment. The draft requirements were augmented with the results of an informal literature search.^12^^–^^14^

Our identified requirements were categorized as either functional (general capabilities, oncology-specific requirements and oncology-specific enhancements) and non-functional (interoperability, security and data privacy). Our team of experts reviewed the initial list of 253 requirements and prioritized 142 items as the most relevant. To account for variations in the needs and resources of the centres, the requirements were categorized as either silver (core requirements), gold (enhanced features) or platinum (advanced features with artificial intelligence tools). These prioritized and categorized requirements were further refined using input from industry experts, electronic records vendors and technical experts in digital health initiatives. Subsequent validation involved interviews with clinicians and health information technology teams from hospitals that had responded to the May 2022 survey, and a virtual workshop delivered by the Koita Centre of Digital Oncology to National Cancer Grid members and information technology and digital health experts. Our final 216 requirements (162 functional and 54 non-functional) are listed in the online respository.^15^ The inclusion of 82 general requirements and their alignment with the digital health standards^16^ published by the National Accreditation Board of Hospitals and Healthcare Providers, India, facilitates the implementation of electronic records products in centres that also treat patients with diseases other than cancer.

### Vendor selection 

We published an invitation to potential suppliers and evaluated submitted bids through a three-step process: prequalification, technical evaluation including product demonstration and financial evaluation. We received bids from 17 vendors, shortlisted 13 and enlisted six different vendors to create electronic medical records products (online repository).^15^ We selected six vendors rather than a single vendor to develop these products to enable competition and better pricing, and to minimize the risk of failure. All selected vendors agreed to follow our developed requirements and the guidelines of the Ayushman Bharat Digital Mission,^11^ adhere to the development timeframe of 18 months, use relevant clinical standards for interoperability, comply with security and data privacy, and provide training and support. 

### Modules and templates

We collated existing best practices and workflows from multiple National Cancer Grid centres, and created draft modules and templates for the different sections of the electronic medical records (e.g. multidisciplinary clinics, staging, oncology specialities, cancer site and subsite, and palliative care). We finalized these modules and templates through an iterative process based on inputs from the core team, various clinical subcommittees (to ensure alignment between modules) and an open stakeholder consultation. Our objective was to develop modules that capture the clinical workflow intuitively, reduce the time spent on documentation and present information in chronological order to assist clinical decision-making.

### Interoperability

A subcommittee comprising core team members, digital health experts and selected vendors created a blueprint to facilitate interoperability, which will be published as digital public goods after pilot testing. The key components included the development of a data dictionary, the identification of relevant data variables for information exchange, the coding of variables following the industry standard Fast Healthcare Interoperability Resources rules and specifications, and the validation of interoperability.

### Key performance indicators 

Our core team and other experts evaluated and finalized a list of key performance indicators identified from the literature. These indicators will be available as summary data and dashboards to relevant stakeholders for quality monitoring and improvement. The methods by which these indicators are measured are described at the relevant data fields.

### Procurement and adoption

To achieve the benefits of our developed electronic medical records products, it is essential to support centres in selecting an appropriate product according to their requirements and resources. To facilitate adoption across centres, our team of technical and digital health experts initiated an assistance programme by assessing centre readiness in terms of leadership commitment and required investment in infrastructure enhancement for product deployment. Our procurement and adoption programme involves technical assistance with selection of the most suitable product, mediating interactions with vendors for product demonstration and negotiating price, and partial funding (applicable for only public and charitable hospitals). We sourced the funding for procurement and adoption through corporate social responsibility grants (online repository).^15^

## Relevant changes

Before inviting vendors to submit a product development bid to develop a new electronic medical records product, we invested much time and effort into developing specific requirements.

Our collaboration between multidomain experts and vendors led to the establishment of six new, high-quality and interoperable products, compliant with the varying needs and resources of hospitals. 

To ensure the implementation of these new products, we supported 20 centres with the procurement and adoption of an electronic medical records product through partial funding and technical assistance. 

## Lessons learnt

Box 1 provides a summary of the main lessons learnt during the development and deployment of six different electronic medical records systems.

Box 1 **Summary of main lessons learnt**

• In developing comprehensive electronic medical records product requirements, we gained an appreciation of multisectoral collaboration and an understanding of the challenges faced by hospitals in implementing such systems.

• By inviting vendors to submit a product development bid, we ensured that the cost of product development was borne by the digital health companies; by working with more than one vendor, we ensured that health centres and patients benefited from competition.

• By monitoring the implementation of the electronic medical records products and incorporating user feedback, we are addressing issues raised by health workers and encouraging adoption of the products.

In developing comprehensive electronic medical records product requirements, we gained an understanding of the challenges faced by hospitals and vendors because of a lack of technical or clinical expertise, respectively. By considering the varying requirements of hospitals with different levels of resources, we have ensured that our initiative can be replicated in other countries. By making these requirements available as digital public goods, we hope to contribute to the improvement in quality of electronic records solutions worldwide.

By inviting vendors to submit a product development bid, we ensured that the cost of product development was borne by digital health solutions companies. By awarding six different vendors with a contract, we benefited National Cancer Grid centres (and therefore patients) by ensuring fair pricing and competition; vendors will have access to the large market of hospitals currently without an electronic medical records system. 

We are aware of resistance to these new systems by clinicians who find it difficult to allocate time for data documentation. To address this problem, we are testing artificial intelligence solutions to digitize health records without the need for manual data entry, avoiding disruptions to interactions between patients and health workers. Other future changes include monitoring the implementation of the electronic medical records products and incorporating user feedback; developing clinical decision support systems and integrating these with electronic records; and using deidentified digital data to enhance cancer registries and promote research.
